# Transmission of Norwegian reindeer CWD to sheep by intracerebral inoculation results in an unusual phenotype and prion distribution

**DOI:** 10.1186/s13567-024-01350-6

**Published:** 2024-07-29

**Authors:** Erez Harpaz, Federico Angelo Cazzaniga, Linh Tran, Tram T. Vuong, Giuseppe Bufano, Øyvind Salvesen, Maiken Gravdal, Devin Aldaz, Julianna Sun, Sehun Kim, Luigi Celauro, Giuseppe Legname, Glenn C. Telling, Michael A. Tranulis, Sylvie L. Benestad, Arild Espenes, Fabio Moda, Cecilie Ersdal

**Affiliations:** 1 Department of Production Animal Clinical Sciences, Norwegian University of Life Sciences, Sandnes, Norway; 2 Unit of Neurology 5 and Neuropathology, Fondazione IRCCS Istituto Neurologico Carlo Besta, Milan, Italy; 3 Section for Biohazard and Pathology, Norwegian Veterinary Institute, Ås, Norway; 4 Prion Research Center (PRC) and the Department of Microbiology, Immunology and Pathology, Colorado State University, Fort Collins, CO USA; 5 Department of Neuroscience, Scuola Internazionale Superiore di Studi Avanzati (SISSA), Trieste, Italy; 6 Department of Preclinical Sciences and Pathology, Norwegian University of Life Sciences, Ås, Norway; 7 Present Address: Åkerblå AS, Haugesund, Norway

**Keywords:** prions, Chronic wasting disease, sheep, Norway, interspecies transmission, reindeer, intracerebral inoculation

## Abstract

Chronic wasting disease (CWD), a prion disease affecting cervids, has been known in North America (NA) since the 1960s and emerged in Norway in 2016. Surveillance and studies have revealed that there are different forms of CWD in Fennoscandia: contagious CWD in Norwegian reindeer and sporadic CWD in moose and red deer. Experimental studies have demonstrated that NA CWD prions can infect various species, but thus far, there have been no reports of natural transmission to non-cervid species. In vitro and laboratory animal studies of the Norwegian CWD strains suggest that these strains are different from the NA strains. In this work, we describe the intracerebral transmission of reindeer CWD to six scrapie-susceptible sheep. Detection methods included immunohistochemistry (IHC), western blot (WB), enzyme-linked immunosorbent assay (ELISA), real-time quaking-induced conversion (RT-QuIC) and protein misfolding cyclic amplification (PMCA). In the brain, grey matter vacuolation was limited, while all sheep exhibited vacuolation of the white matter. IHC and WB conventional detection techniques failed to detect prions; however, positive seeding activity with the RT-QuIC and PMCA amplification techniques was observed in the central nervous system of all but one sheep. Prions were robustly amplified in the lymph nodes of all animals, mainly by RT-QuIC. Additionally, two lymph nodes were positive by WB, and one was positive by ELISA. These findings suggest that sheep can propagate reindeer CWD prions after intracerebral inoculation, resulting in an unusual disease phenotype and prion distribution with a low amount of detectable prions.

**Supplementary Information:**

The online version contains supplementary material available at 10.1186/s13567-024-01350-6.

## Introduction

Prion diseases are a group of fatal, neurodegenerative disorders characterized by the conformational conversion of the cellular prion protein (PrP^C^) into a pathological form known as PrP^Sc^. These diseases propagate through the amplification of disease-associated PrP^Sc^, or prions, in a templated manner [1]. They may manifest as spontaneous (often referred to as sporadic), acquired, or genetic diseases [2] and can occur in humans or animals [3]. In animals, classical scrapie in sheep and goats and classical chronic wasting disease (CWD) in cervids are contagious prion diseases that spread horizontally among hosts. Classical scrapie typically occurs in local outbreaks within flocks [4], while contagious CWD is endemic among wild animals in various North American (NA) regions [5]. Susceptibility to prion diseases is influenced by polymorphisms in the prion protein-encoding gene (*PRNP*). In sheep, the most susceptible *PRNP* allele is valine at position 136, arginine at position 154, and glutamine at position 171, collectively known as VRQ. On the other hand, the presence of alanine at position 136 and arginine at positions 154 and 171 (ARR) confers resistance to classical scrapie [6, 7]. It is important to note that while arginine at position 154 is present in both susceptible (VRQ) and resistant (ARR) alleles, the combination of residues at these positions determines overall susceptibility or resistance to disease.

Contagious CWD has been recognized in the United States for more than 50 years [8]. In 2016, the first European case was found in a Norwegian wild reindeer in the Nordfjella mountain area [9], followed by 18 other CWD-positive animals in the same area and two additional cases on the Hardangervidda plateau, located south of Nordfjella [10–12]. Furthermore, geographically dispersed cases of CWD have been detected in moose and red deer in Norway [13, 14], as well as in moose in neighboring Sweden and Finland [11, 15]. The forms of CWD observed in moose and red deer appear atypical in comparison to the contagious form, with sporadic occurrence in old animals and prions being confined to the brain [15]. In contrast, CWD in reindeer affects young adults with certain *PRNP* genotypes [16] and exhibits significant involvement of peripheral lymphatic organs [11]. Subsequent analyses of these prion isolates from reindeer, moose, and red deer have revealed that the recently identified CWD forms in Fennoscandia can be attributed to at least four distinct prion strains, all of which are different from the known NA CWD strains [17–22].

Since available data indicate that reindeer CWD is contagious [12] and evidence from the NA situation suggests that affected animals can shed prions to the environment [23, 24], a risk of spillover to other cervid species and grazing livestock exists. During summer, Norwegian sheep farmers commonly transport their sheep to high-quality mountain pastures. One such area is the CWD-affected Nordfjella, which hosts approximately 70 000 sheep each summer. In this area, spatiotemporal interactions between sheep and reindeer have been documented, including overlapping movements between a diseased CWD reindeer and grazing sheep [25, 26]. Although the incidence of reindeer CWD in Norway seems low, the capacity for prions to survive in environmental reservoirs such as soil, plants and water raises concerns about possible indirect interspecies transmission [27–29].

NA CWD strains have been experimentally transmitted to sheep, although with low efficacy [30–32]. Currently, no in vivo data exist on the potential transmission of reindeer CWD to sheep. In this paper, we describe the experimental transmission of reindeer CWD to sheep and further characterize the resulting disease manifestation. Lambs with the classical scrapie-susceptible *PRNP* genotype VRQ/VRQ were intracerebrally inoculated with reindeer CWD. These animals were monitored for up to 42 months post-inoculation via regular neurological examinations and sampling of rectoanal mucosa-associated lymphoid tissue (RAMALT). At the study endpoint, a comprehensive postmortem examination was performed, and a variety of prion detection techniques were employed, including the highly sensitive seed amplification assays real-time quaking induced conversion (RT-QuIC) and protein misfolding cyclic amplification (PMCA).

## Materials and methods

### Animals

Six three-month-old female lambs (Table 1), all with the VRQ/VRQ *PRNP* genotype, were selected from a research flock at the Section of Small Ruminant Research and Herd Health, Department of Production Animal Clinical Sciences, Norwegian University of Life Sciences in Sandnes, Norway. The animals were housed in an isolated facility that complied with the standards approved by the Norwegian Food Safety Authority and subjected to a 16-h light/8-h dark cycle. They were fed hay and commercial pellet concentrate provided twice daily, with continuous access to water. Additionally, tissues from one non-inoculated VRQ/VRQ sheep belonging to the research flock and one sham inoculated VRQ/VRQ sheep [33] served as negative controls.

**Table 1:** **Timing of euthanasia and clinical signs**

Animal	Time of euthanasia (mpi)	Reason for euthanasia	Clinical signs or disease prior to euthanasia
90530	17	Intercurrent disease	Lethargy, clinical hypocalcemia
90542	31	Intercurrent disease	Degenerative joint disease
90501	42	Study endpoint	Spontaneous smacking, reduced menace response
90506	42	Study endpoint	Abnormal mental status, spontaneous smacking, reduced menace response, positive scratch test, abnormal gait
90507	42	Study endpoint	Reduced menace response
90525	42	Study endpoint	Spontaneous smacking, teeth grinding, reduced menace response, positive scratch test, abnormal gait

*mpi* months post-inoculation.

### Inoculum and inoculation procedure

The inoculum was a pool of two CWD-positive reindeer brains (ID 17-CD11087, S_225_/Y_225_ *PRNP* genotype; ID 18-CD2788, S_225_/Del_84-91_ *PRNP* genotype). Both donors were derived from the Nordfjella outbreak, and one of the donors was characterized earlier [19]. The inoculum was primarily diluted in sterile water to a 20% w/v solution and then further diluted with 5% sterile glucose to a 10% w/v solution. Streptomycin and penicillin were added to achieve a final concentration of 1% for each of the antibiotics.

For the inoculation process, the lambs were fully anaesthetized with a tiletamine and zolazepam mixture supplemented with lidocaine/adrenaline as local subcutaneous anaesthesia. A midline skin incision was made at the junction of the parietal and frontal bones, and a 1-mm hole was drilled through the calvarium. The inoculum (0.5 mL) was injected using a 5 cm, 21-gauge cannula with gradual withdrawal of the needle as the inoculum was deposited. The skin was closed with two sutures, and flunixin was given to provide post-operative analgesia. Crystallized penicillin was given intravenously immediately after the procedure, and the animals were additionally treated for 3 days with benzylpenicillin and for 5 days with tetracycline. A table detailing the anaesthesia and treatment regimen is provided in Additional file 1.

### Clinical evaluation and sampling

*RAMALT* biopsies were sampled twice a year starting at 12 months post-inoculation (mpi) and subsequently at 18, 24, 30, and 36 mpi. Each animal was manually restrained, and the rectal mucosa was topically anaesthetized with 2% lidocaine gel. A lubricated, disposable RAMALT biopsy rectal speculum (Veterinary Instrumentation, Shefield, UK) was inserted into the rectum. Biopsies were taken from the ventral, right lateral or left lateral rectal wall. The mucosa was gently retracted using sterile forceps approximately 1 cm proximal to the mucocutaneous junction and incised using sterile surgical scissors to produce an elliptic tissue sample. The speculum was then retracted from the rectum, and the sheep were observed for signs of bleeding or discomfort. Each biopsy sample was divided vertically; one half was fixed in 4% formaldehyde solution, and the other half was frozen immediately at – 70 ℃ for future analysis. No complications occurred following the biopsies.

*Clinical and neurological examinations* were performed every other month, or when necessary, by two veterinarians. The neurological examination included the evaluation of cranial nerve functions, pruritus (scratch test and wool examination) and gait. In the case of intercurrent disease, a full clinical examination supplemented by ancillary tests was performed. Blood panels, including serum copper levels, were analysed in house (Atomic Absorption spectrometer AAnalyst 300, Perkin Elmer, CT, USA) when necessary.

### Post-mortem tissue collection

Euthanasia was performed by intravenous injection of an overdose of pentobarbital and was immediately followed by complete necropsy. Samples were collected from various tissues, including the brain and spinal cord with dorsal root ganglia, the trigeminal ganglion, the cranial mesenteric and coeliac ganglion, and several lymphatic tissues. Samples for histological analysis were preserved in 4% formaldehyde solution for approximately 10 days, dehydrated in graded ethanol and xylene, and embedded in paraffin. Tissue samples for other analyses were immediately frozen at – 70 ℃. A liver sample from each animal was sent to the Institute of Marine Research, Bergen, Norway, for analysis of trace elements.

### Histopathology and immunohistochemistry

Three-micron-thick tissue sections were placed on either plain glass slides (histopathology) or on coated slides for immunohistochemistry (IHC) (Superfrost Plus^®^, Menzel-Gläser, Thermo Scientific, Braunschweig, Germany). One half of the brain was examined at seven different levels: the olfactory lobe, the frontal cortex at the ansate sulcus, transverse through the piriform lobe and thalamus, including the hippocampus, transverse through the superior colliculus, sagittal at the midline of the cerebellum, transverse through the cerebellum and brainstem at the caudal cerebellar peduncle and transverse at the obex. The spinal cord was sectioned at each vertebral segment and at the cauda equina. The dorsal root ganglia were included if present. Peripheral tissues included were the trigeminal ganglion, cranial mesenteric and coelic ganglion, parotid (PLN), medial retropharyngeal (RPLN), superficial cervical (SCLN), jejunal (JLN), and distal jejunal (DJLN) lymph nodes, palatine tonsils, tonsils of the soft palate, spleen, and gut-associated lymphatic tissue (GALT). The extent of vacuolation in defined areas of the brain and spinal cord was evaluated at 10X magnification. Vacuolation was graded as follows: 1–2 vacuoles: scored 0.5; < 5 vacuoles: scored 1; 6–15 vacuoles: scored 2; and > 15 vacuoles: scored 3.

IHC was mainly performed with the anti-PrP antibodies F89/160.1.5 directed against residues 142–154 on the ovine prion protein (Thermo Scientific, Braunschweig, Germany), diluted 1:2000 [34] and F99.97.6.1 directed against residues 220–225 (VMRD, WA, USA), diluted 1:800 [35]. Glial fibrillar acidic protein was labelled (GFAP, Z0334, Agilent Dako, CA, USA), and a 1:2000 dilution was used for labelling of selected brain and spinal cord sections. Slides were deparaffinized and then subjected to 15 min of immersion in 98% formic acid, followed by hydrated autoclaving in citrate buffer (pH 6) at 121 ℃ for 15 min. These two antigen retrieval steps were omitted from the GFAP procedure. After cooling, endogenous peroxidase activity was suppressed by a 15-min incubation with a 3% H_2_O_2_ solution diluted in methanol. To block non-specific binding sites, the slides were exposed to 1:50 normal goat serum diluted in 5% bovine serum albumin (BSA) for 20 min. The membranes were incubated with primary antibodies for 1 h at room temperature. The subsequent steps were performed either by the Envision + system for anti-PrP antibodies (K4005, Agilent Dako, CA, USA) or by the MACH 1 Universal HRP Polymer Detection kit for GFAP (M1U539, Biocare Medical, CA, USA). To visualize the signals, the samples were immersed in a solution of 3-amino-9-ethylcarbazole (AEC) for 8–10 min and counterstained with hematoxylin for 1 min. Each run included a scrapie-positive, a scrapie-negative, and a methodological control, where the primary antibody was substituted with 1% BSA.

### Sample preparation for RT-QuIC and PMCA analyses

Frozen samples from the RAMALT, brain (olfactory bulb, frontal cortex, hippocampus, midbrain/diencephalon, cerebellum and distal medulla oblongata), spinal cord (cervical, thoracic and lumbar), lymph nodes (RPLN, PLN, SCLN, and DJLN), and spleen (only for the PMCA) were homogenized at 10% w/v in lysis buffer (100 mM sodium chloride, 10 mM ethylenediaminetetraacetic acid tetrasodium salt (EDTA), 0.5% Nonidet P-40, 0.5% sodium deoxycholate, and 10 mM Tris, pH 7.4). RAMALT (RH), brain (BH), spinal cord (SCH) and lymph node homogenates (LnH) were then centrifuged at 4 ℃ and 800 *g* for 1 min to remove cellular debris. The supernatant was then subjected to different analyses. BH, SCH, and LnH were each diluted 10^–3^ in phosphate-buffered saline (PBS) (volume/volume, v/v) before RT-QuIC and PMCA analyses. Six microliters of this dilution was used to perform RT-QuIC analysis, and 10 μL of each sample of RH, BH, SCH and LnH was subjected to PMCA analysis.

### RT-QuIC recombinant substrate production

The pet11a plasmid encoding the truncated bank vole prion protein (BvPrP_90-231_, GenScript) was transformed into *Escherichia coli* BL21 (DE3) cells (New England Biolabs, MA, USA). The cells were grown overnight in Luria Bertani (LB) medium supplemented with 100 μg/mL ampicillin. Protein expression was induced by the addition of 0.75 mM isopropyl b-D galactopyranoside (IPTG), and the cells were incubated for 12 h at 37 ℃. The cells were homogenized (PandaPLUS 2000), and the extracted inclusion bodies were washed in bi-distilled water several times and dissolved in 8 M guanidine hydrochloride (Gnd-HCl). The protein was purified by size exclusion chromatography (HiLoad 26/60 Superdex 200-pg column, Cytiva, MA, USA) in 5 M GndHCl, 25 mM Tris–HCl (pH 8), and 5 mM EDTA. The protein-containing fractions were analysed by sodium dodecyl sulfate‒polyacrylamide gel electrophoresis (SDS-PAGE). Protein refolding was performed by dialysis in refolding buffer (20 mM sodium acetate and 0.005% NaN3, pH 5.5) using a 3.5 kilodalton (kDa) membrane (Spectrapor, AZ, USA). The protein was then dialyzed in PBS (pH 5.8), quantified and stored at – 80 ℃. All salts used were obtained from Sigma‒Aldrich, MO, USA.

### RT-QuIC procedure

Truncated recombinant prion protein (recPrP) with the bank vole amino acid sequence (BvPrP_90-231_) was thawed at room temperature and then filtered through a 100 kDa Nanosep centrifugal device (Pall Corporation, NY, USA). Subsequently, the samples were analysed in triplicate using a black 96-well optical flat-bottom plate (Thermo Scientific, Braunschweig, Germany). The final reaction volume was 100 µL, and the reagents (Sigma‒Aldrich, Mo, USA) were 150 mM NaCl, 0.002% SDS, 10 mM PBS, 1 mM EDTA, 10 µM thioflavin-T (ThT), and 0.135 mg/ml of recPrP. To prevent contamination, reaction mixtures were meticulously prepared and loaded (98 µL) into each well of the microplate in a prion-free laboratory. Following the addition of 2 µL per replicate of diluted BHs, SCHs and LnHs (10^–3^), the plate was securely sealed with a film (Thermo Scientific, Braunschweig, Germany) and inserted into a FLUOstar OMEGA microplate reader (BMG Labtech, Ortenberg, Germany). The incubation occurred at 55 ℃, with alternating cycles of 1 min shaking (at 600 rpm, double orbital) and 1 min incubation. Fluorescence readings (480 nm) were recorded at 15-min intervals (30 flashes per well at 450 nm).

To determine positive seeding activity, a threshold of 110 h was set for BH and SCH, and a threshold of 40 h was set for LnH. These thresholds were determined using reference data from the respective tissue derived from healthy control sheep. A sample was considered positive when at least 2 out of 3 replicates crossed the fluorescence threshold set at 10 000 arbitrary units (AU) before reaching the time threshold. The data are visually presented in diagrams showcasing the time taken for each replicate (indicated by black dots) to reach the fluorescence threshold (lag phase).

### Preparation of the PMCA substrate

The frontal cortex of a healthy sheep with the VRQ/VRQ *PRNP* genotype was homogenized at 10% (w/v) in conversion buffer (1 × PBS containing 150 mM sodium chloride and 1% Triton X-100) supplemented with protease inhibitors cocktail (Roche, Basel, Switzerland) and used as a PMCA reaction substrate. We have recently shown that reindeer CWD is amplified in the brains of sheep with both the VRQ/VRQ and ARQ/ARQ *PRNP* genotypes [22]. To increase the PMCA efficiency, 0.1 mg/mL heparin, 0.05% digitonin, and 3 polytetrafluoroethylene (PTFE) beads were used to supplement the substrate.

### PMCA analysis

Ten microliters of the RH, BH, SCH and LnH samples, which were prepared as previously described, were added to 90 μL of the PMCA substrate. This mixture was then transferred into 0.2 mL PCR tubes and subjected to amplification using a Qsonica Q700 sonicator (Qsonica, NY, USA). The PMCA process involved alternating cycles of incubation (29 min and 40 s) and sonication (20 s) at a potency of 150–170 Watts. After 48 h of reaction, which was considered one round of amplification, 10 μL of the amplified material was transferred to 90 μL of freshly prepared PMCA substrate to initiate another round of amplification. This process was repeated for a total of 6 PMCA rounds for each sample. To maintain stringent prion-free conditions and prevent contamination, PMCA substrates were prepared under strict conditions and in a prion-free laboratory. Additionally, the horn of the sonicator was periodically decontaminated with 4 M Gdn-HCl overnight. Appropriate negative controls were included in each PMCA round to monitor for any contamination or de novo generation of prions. No contamination or de novo prion formation was observed throughout the experimental process.

### Western blotting

Ten microliters of PMCA-generated RH, BH, SCH and LnH products were treated with 50 μg/mL proteinase K (PK, Invitrogen, MA, USA) for 1 h at 37 ℃ under shaking (550 rpm), mixed with loading buffer (Bolt^™^ LDS Sample Buffer 4X and DTT 10X, Thermo Scientific, MA, USA), and boiled for 10 min. The products were then loaded onto 12% Bis–Tris plus gels (Thermo Scientific, MA, USA) and subjected to electrophoresis analysis under denaturing conditions (SDS-PAGE). Following electrophoresis, proteins were transferred onto polyvinylidene difluoride (PVDF) membranes (Merck Millipore, Darmstadt, Germany) and blocked with nonfat dry milk (Santa Cruz Biotechnology, TX, USA) for 1 h at room temperature. The PVDF membranes were probed with the monoclonal anti-PrP antibody 6D11, diluted 1:5000 (Covance, NC, USA), followed by incubation with whole anti-mouse IgG conjugated with horseradish peroxidase (HRP). The blots were visualized using a chemiluminescence system (ECL Prime, GE Healthcare Amersham, UK).

The WB method used directly on the midbrain, spinal cord, PLN, RPL, DJLN, SCLN and spleen from the inoculated animals was performed using a commercial kit (TeSeE, Bio-Rad, CA, USA) and described in detail earlier [22]. The samples were digested by PK provided in the kit prior to gel electrophoresis (NuPAGE 12% Bis–Tris protein gels, Thermo-Fisher, MA, USA). The proteins were transferred onto PVDF membranes using a trans-blot turbo system (Bio-Rad, CA, USA) and were then further labelled with the Sha31 antibody provided by the manufacturer. The signal was visualized using an Azure c280 (Azure Biosystems, CA, USA).

### Enzyme-linked immunosorbent assay

Samples from the midbrain, obex, spinal cord, PLN, SCLN, RPLN, DJLN and spleen were analysed with a TSE commercial kit (IDEXX HerdChek BSE/Scrapie, ME, USA) as described in detail earlier [26]. One hundred and twenty microliters of each homogenized sample (20% w/v) was mixed with 30 μL of diluent solution. Then, 100 μL of this mixture was placed into the wells of the antigen capture plate and incubated for 45 to 60 min on a shaking machine. After washing, the plate was incubated with conditioning buffer for 10 min, conjugated with an anti-PrP antibody for 45 min, and then 3,3′,5,5′-tetramethylbenzidine was added for 15 min in the dark. A stop solution was added before the optical density (OD) of each sample was read using a spectrophotometer at 450 and 620 nm. To establish the cut-off value for each test, the average OD of the two negative controls was calculated, and a constant factor of 0.120 (BSE), 0.150 (CWD) or 0.180 (scrapie) was added.

### Mouse bioassay

Homogenates of the RPLN and midbrain from sheep 90530 (euthanized at 17 mpi) were intracerebrally inoculated into gene-targeted (Gt) mice expressing elk or deer PrP, referred to as GtE and GtQ, respectively, and into transgenic (Tg) mice expressing different sheep scrapie susceptibility alleles, varying only at a single amino acid at PrP residue 136. GtE and GtQ mice were previously generated [36] using a homologous recombination strategy in embryonic stem (ES) cells from FVB mice in which the mouse PrP coding sequence of *Prnp* was replaced with the corresponding elk or deer PrP coding sequence. The brains of GtE and GtQ mice express equivalent amounts of elk and deer PrP at the same level as those found in the brains of wild-type FVB mice. Tg mice expressing ovine PrP with alanine (A) at (OvPrP-A136) or valine (V) (OvPrP-V136) at this position have also been described previously [37] and are referred to as Tg(OvPrP-A136)3533^±^ and Tg(OvPrP-V136)4166^±^ mice, respectively. Tg mice express the OvPrP coding sequence, except for the addition of an extra glycine residue at codon 31 and the addition of the mouse PrP N-terminal signal peptide instead of the OvPrP signal peptide. Tg mice were generated by cloning these OvPrP-A136 and OvPrP-V136 expression constructs into the cosSHa Tet cosmid vector [38] and microinjection of embryos from inbred Prnp^0/0^/FVB mice. Both lines were maintained with the transgene in the hemizygous state, resulting in CNS expression similar to that found in the brains of wild-type FVB mice.

The inoculated groups consisted of ten animals, five of each sex. The mice were followed for 500–600 days post-inoculation for clinical signs. Following euthanasia, the brains and spleens of the mice were examined using the previously described methods of IHC and histoblotting [18] and by WB using D18 as the primary antibody [39]. Furthermore, spleens from the mice were examined by RT-QuIC using the Syrian hamster RecPrP_90-231_ as a substrate. The samples were analysed in quadruplicate, and 2 µL of homogenate was added to 98 µL of the reaction mixture and loaded into a black 96-well optical flat bottom plate (Thermo Scientific, MA, USA). The final reaction substrate volumes included 170 mM NaCl, 0.002% SDS, 10 mM PBS, 1 mM Syrian hamster RecPrP_90-231_ and 0.01 mM ThT. The samples were analysed via a FLUOstar OMEGA microplate reader (BMG Labtech, Ortenberg, Germany) with the following parameters: incubation at 42 ℃, 15-min cycles alternating from 1 min of shaking at 700 rpm (double orbital), and 1 min of rest. Fluorescence readings (480 nm) were recorded at 15-min intervals (20 flashes per well at 450 nm). Runs were allowed for 62 h for a total of 248 cycles, and positivity was determined based on a threshold set at 5 times the standard deviation in arbitrary units (AU) of reference uninfected spleens from the mouse background. Positivity was determined by having 3 out of the 4 replicates crossing the set threshold.

## Results

### Clinical evaluation

During the experiment, all sheep maintained good to high body condition scores, fluctuating between 3.5 and 4.5 points on a 1–5 scale for each sheep. Two sheep were euthanized before the study endpoint due to intercurrent disease. Sheep 90530 was diagnosed with hypocalcemia after shearing and was euthanized 17 months post-inoculation (mpi). Sheep 90542 suffered pain and lameness due to arthrosis in the right elbow joint and was euthanized at 31 mpi. In the period from 38 mpi to the study endpoint at 42 mpi, the remaining four sheep developed inconsistent neurological signs (Table 1).

### Ante-mortem RAMALT biopsies

In an attempt to detect peripheral dissemination of prions during the preclinical phase, RAMALT biopsies were sampled every 6^th^ month starting at 12 mpi. All the samples were negative according to the IHC results (data not shown). PMCA analysis of these samples was also negative (Additional file 2).

Histologically, there was no apparent correlation between the number of lymphoid follicles in the RAMALT samples and the age of the animals. The highest average follicular yield was in the first biopsy taken at 12 mpi, the second highest was in the final biopsy taken at 36 mpi, and the lowest yield was found at 18 mpi. Fourteen of the 24 available RAMALT samples contained more than 5 follicles, and only one sample contained no follicles (Additional file 3).

### Brain and spinal cord

**Figure 1:**
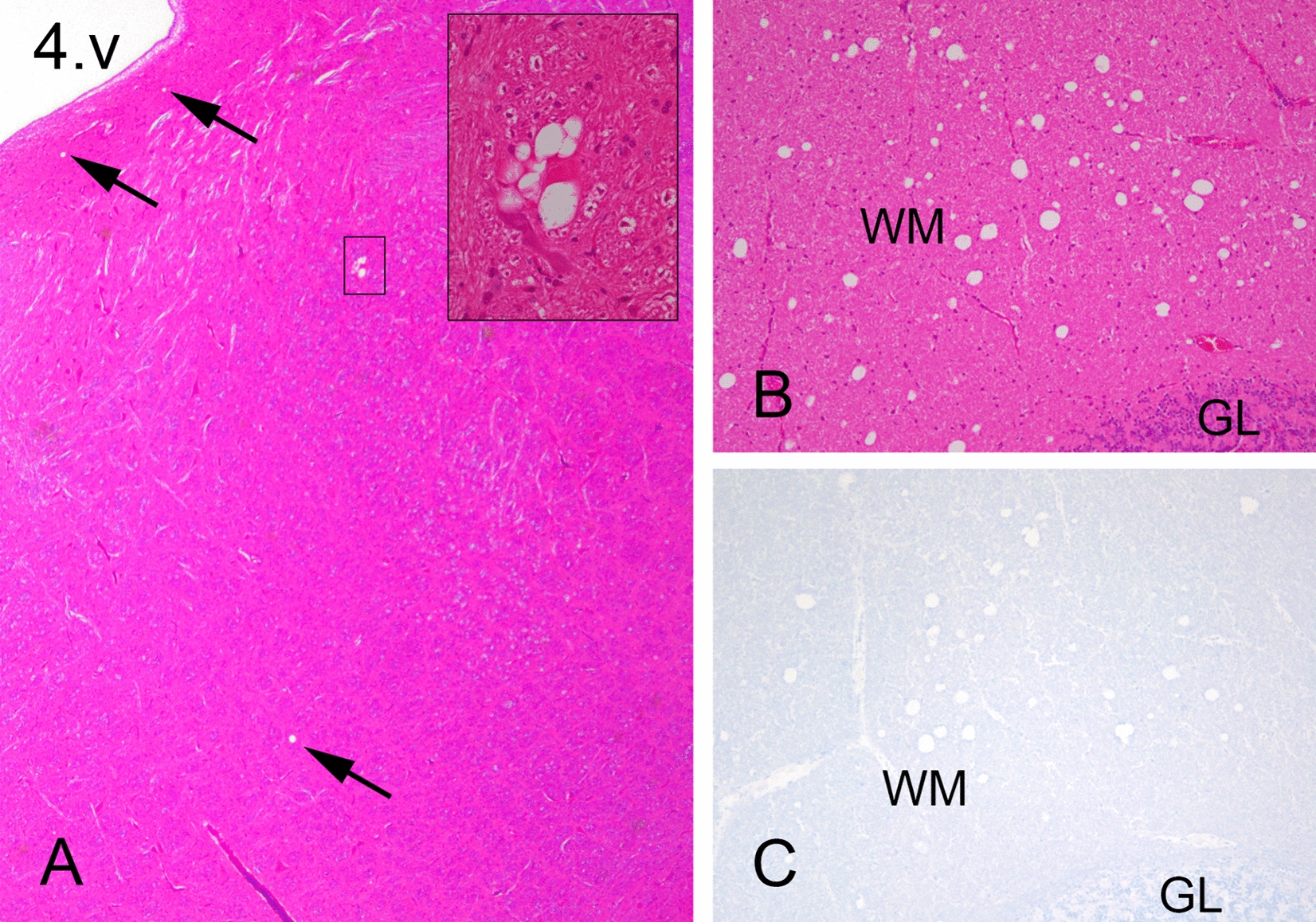
**Sparse and scattered vacuoles in the grey matter and unusual vacuolation in the white matter**.** A** There are few and scattered vacuoles (arrows) in the reticular formation and medial vestibular nucleus in the brainstem at the level of the *radix nervi facialis* of sheep 90525. The marked inset shows a close-up image of a group of vacuoles next to a neuron. 4. v: fourth ventricle. **B** There are many vacuoles in the medial white matter of the cerebellum of sheep 90506. **C** There was no PrP immunolabelling associated with vacuoles in the cerebellar white matter. *WM* white matter, *GL* granule cell layer. A, 25 × magnification; inset, 400 × magnification; HE, 100 × magnification; B, 100 × magnification; C, 100 × magnification; F89, monoclonal antibody.

Most sheep showed a few scattered vacuoles associated with grey matter neuropil in some or all of the following regions: the medulla oblongata, pons, mesencephalon, and thalamus (Figure 1A). Notably, all inoculated sheep exhibited white matter vacuolation in several brain regions to some degree. In particular, sheep 90506 showed marked vacuolation in the central white matter of the cerebellum (Figures 1B and C). The corpus callosum, capsula interna and tractus opticus contained variable degrees of vacuolation in all sheep. Detailed vacuolation scores are provided in Additional file 4. All animals showed varying degrees of degenerative changes, including spheroids and digestive chambers, and increased astrocytic activity in the white matter of the spinal cord. These changes were different from the changes typically present in prion disease. Comparable changes were previously observed in one sheep experimentally infected with NA CWD but judged to be unrelated to prion disease [30]. None of the examined brain or spinal cord sections were positive using the F89 or F99 anti-PrP antibodies (Figure 1C). In addition, WB and ELISA results for selected areas were negative (data not shown). To exclude mineral deficits, particularly copper (Cu) deficiency, which could be a potential cause of the described histological lesions, the liver from each animal was analysed for a comprehensive list of trace elements. There were no major findings in the liver analysis, and the serum Cu concentration was within the normal range for all sheep. The results of these analyses are listed in Additional file 5.

**Figure 2:**
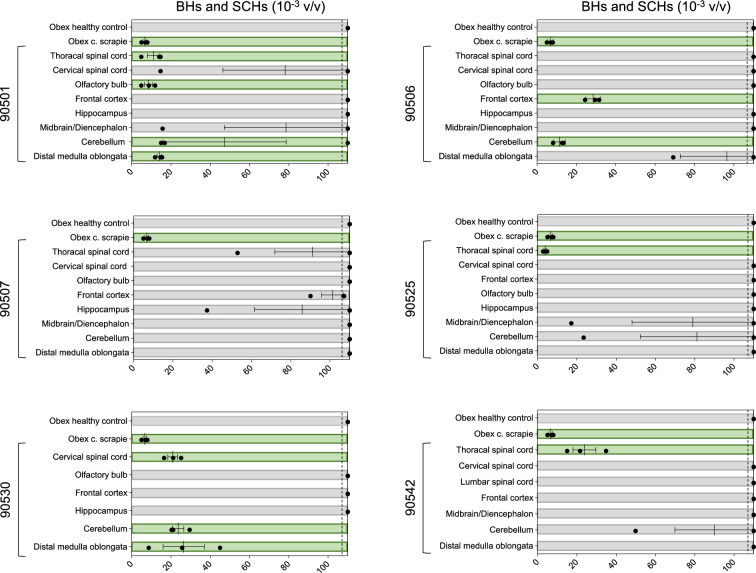
**Real-time quaking-induced conversion (RT-QuIC) on brain and spinal cord samples**. Brain (BH) and spinal cord (SH) homogenates were diluted to 10^–3^ volume/volume (v/v), and 2 μL was subjected to RT-QuIC analysis using recombinant BvPrP_90-231_ brain as substrate. Each sample underwent triplicate analysis, with black dots denoting the time for each replicate to reach the fluorescence threshold (lag phase). The dotted lines show the selected time point (110 h) to define the positive (green box) and negative (grey box) samples. The mean value and standard error of the mean (SEM) are indicated. The obex homogenate from a healthy control sheep was diluted to 10^–3^ (v/v) in PBS and used as a negative control. The obex homogenate from a sheep inoculated with classical scrapie (c. scrapie) was diluted to 10^–3^ (v/v) and used as a positive control.

**Figure 3:**
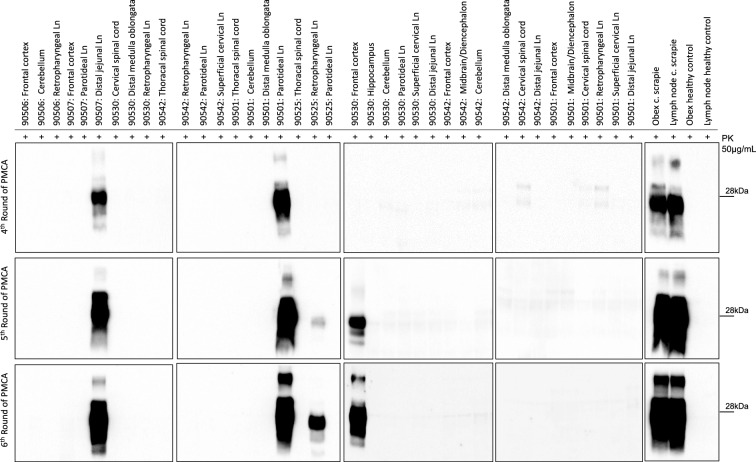
**Analysis of brain, spinal cord and lymph node homogenates by protein misfolding cyclic amplification (PMCA)**. Western blots of the 4th-6th PMCA rounds are shown. After 6 rounds of amplification, PrP^Sc^ was detected with the monoclonal antibody 6D11 in 5 out of 65 samples acquired from the inoculated sheep. Thirty-eight of these samples are shown here. Samples from healthy controls and from sheep subjected to classical scrapie were used as controls. All amplified PrP^Sc^ were characterized by a predominant di-glycosylated band. The numbers on the right of each WB indicate the molecular weight marker in kilodaltons (kDa). The amplified sample from the thoracic spinal cord of sheep 90506 is not shown but can be seen in Additional file 6, along with the remaining wells that are not included in this figure. Ln: lymph node. PK: proteinase K. C. Scrapie: classical scrapie.

To assess prion seeding activity in brain and spinal cord samples, BHs and SCHs were tested using RT-QuIC to determine its impact on the kinetics of the PrP substrate (Figure 2). We decided to utilize recombinant BvPrP_90-231_ because of its high specificity and sensitivity for detecting Norwegian CWD prions [40]. Evaluation of the overall performance of RT-QuIC relied on brain samples obtained from sheep inoculated with classical scrapie and brain samples from healthy animals serving as negative controls. Three sheep (90530, 90501 and 90506) exhibited early protein aggregation in several brain areas—all three in the cerebellum and two in the distal medulla oblongata. Three sheep exhibited protein aggregation by RT-QuIC in the spinal cord, two of which (90542 and 90525) showed seeding activity in the thoracic spinal cord only and not in any other CNS area. Interestingly, one animal (90507) showed no seeding activity in any of the examined CNS areas. Subsequently, the same samples were subjected to PMCA (Figure 3 and Additional file 6), and prions were amplified from the frontal cortex of sheep 90530 and the thoracic spinal cord of sheep 90506. Notably, the amplified products were characterized by a dominant di-glycosylated band. The summarized results, including the RT-QuIC, PMCA, WB and ELISA results, are shown in additional file 7.

### Lymphatic system

**Figure 4:**
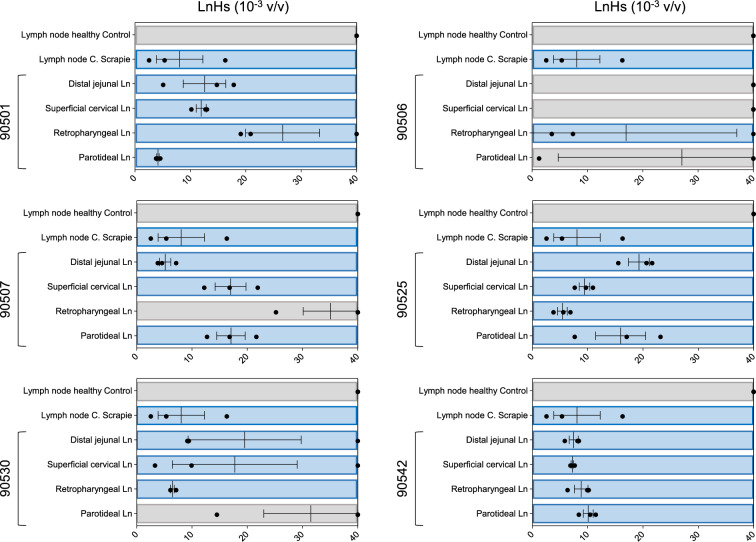
**Real-time Quaking Induced Conversion (RT-QuIC) performed on lymph node (LnH) samples**. Lymph node homogenates (LnH) were diluted to 10^–3^ volume/volume (v/v), and 2 μL was subjected to RT-QuIC analysis using recombinant BvPrP_90-231_ as the substrate. Each sample underwent triplicate analysis, with black dots denoting the time for each replicate to reach the fluorescence threshold (lag phase). The blue boxes represent positive results, while the grey boxes represent negative results. The mean value and standard error of the mean (SEM) are indicated. LnH from healthy control sheep was diluted to 10^–3^ (v/v) and used as a negative control. LnH from sheep inoculated with classical scrapie (c. scrapie) was diluted to 10^–3^ (v/v) and used as a positive control.

Robust RT-QuIC seeding activity was observed in several lymph nodes from all six animals (Figure 4). PMCA successfully amplified PrP^Sc^ in three lymph nodes from three different sheep: (i) the DJLN of sheep 90507, (ii) the PLN of sheep 90501 and (iii) the RPLN of sheep 90525 (Figure 3). Again, the amplified products of PMCA were characterized by a biochemical profile with a predominant diglycosylated band. Remarkably, the DJLN of sheep 90507 and the PLN of sheep 90501 were found to be positive by the conventional WB technique (Additional file 8), in addition to PMCA (Figure 3). Furthermore, the RPLN of 90542 was positive by ELISA (CWD cut-off). These findings corroborated the results obtained from the PMCA and RT-QuIC experiments. The PLN and SCLN of sheep 90525 exhibited borderline results with OD values just below the cut-off in the ELISA test (Additional file 7) but showed seeding activity by RT-QuIC. IHC did not reveal any prion deposition in the examined lymphatic tissues (data not shown).

### Mouse bioassay of sheep 90530

Sheep 90530 were euthanized early in the study (17 mpi), and RPLN and midbrain homogenates from this sheep were intracerebrally inoculated into the mice. None of the four different mouse lines (GtQ226, GtE226, TgARQ and TgVRQ) showed clinical signs consistent with prion disease, and at the study endpoint 500–600 days post-infection, none were positive for PrP^Sc^ by IHC, WB, histoblot or RT-QuIC (data not shown).

## Discussion

In this report, we present the primary transmission of reindeer CWD to sheep through intracerebral inoculation. The sheep selected for this study carried the classical scrapie-susceptible VRQ/VRQ *PRNP* genotype. Prions were amplified in several regions of the brain and spinal cord and in different lymph nodes by the ultrasensitive RT‒QuIC and PMCA techniques. In addition, a few lymph nodes were PrP^Sc^ positive according to standard diagnostic approaches (ELISA and WB). These results indicate that a low amount of prions were present in the tissue samples.

In similar experiments using NA mule deer CWD, prions accumulated both in the CNS and lymphatic tissues of the inoculated sheep but were detected by conventional techniques, suggesting a generally higher PrP^Sc^ load in the examined tissues than in the present study [30, 32]. This discrepancy could be explained by differences in the infectivity of the inoculates, strain properties, or *PRNP* genotypes of the recipient sheep. On the other hand, a direct comparison between NA mule deer and reindeer CWD with PMCA on ovinized ARQ-mouse brain substrate indicated stronger conversion activity by the reindeer prions [21]. In a recent paper from our group, we showed that the reindeer CWD isolate had similar conversion activity by PMCA in VRQ/VRQ and ARQ/ARQ substrates, which could indicate a similar susceptibility for these two *PRNP* genotypes [22].

Prions may show variable tissue tropism upon interspecies transmission [41]. When NA CWD was intracerebrally inoculated into ovine VRQ-transgenic mice (tg338), only a few animals had detectable prions in the brain, but nearly all animals accumulated prions in the spleen, suggesting lymphotropism [42]. This could be attributed to properties of the host in determining prion tissue tropism. Accordingly, cattle show limited accumulation of prions in lymphatic tissues, even when they are inoculated with more lymphotropic scrapie [43] or CWD [44] prions. Sheep, on the other hand, do accumulate C-BSE prions from cattle in their lymphatic system following oral challenge, demonstrating the influence of the host on tissue tropism [45].

Specifically, host *PRNP* influences peripheral prion dissemination, or lymphotropism, as exemplified in sheep scrapie studies [46, 47]. These findings show that susceptible genotypes, such as VRQ/VRQ, exhibit considerable prion accumulation in peripheral tissues following oral and intracerebral inoculations, in contrast to other less susceptible genotypes, such as ARQ/ARR and VRQ/ARR. In an intracerebral inoculation study combining a lymphotropic strain (scrapie) and a host (white-tailed deer), early prion accumulation was observed 7 months post-infection in lymphatic tissues, with no corresponding findings in the brain. At later stages, prions disseminated in both the brain and peripheral tissues [48], which aligns with our observations.

Interestingly, prions were amplified in several CNS areas of sheep 90530 euthanized 17 months after inoculation. The site of inoculation makes it possible that parts of the inoculum were injected into the lateral ventricle and could be spread by cerebrospinal fluid to other parts of the CNS. The remnants of the inoculum might have amplified in these areas.

The four sheep that survived to the study endpoint exhibited variable and inconsistent neurological clinical findings, including some of the signs of prion disease, but still differed from those of scrapie [33, 49] and CWD [8, 50]. Furthermore, we observed aberrant and modest neuropathological changes without detection of prions in the CNS by conventional techniques (IHC, WB and ELISA). However, PrP converting activity was detected in the CNS by ultrasensitive amplification methods. A recent paper detailing the transmission of atypical/Nor98 scrapie to cattle by intracerebral inoculation [51] shares certain similarities with our study: prions were not detected in the brain through WB or IHC but were amplified by PMCA. However, findings in peripheral tissues were not reported. The results of the mouse bioassays were also negative in both studies. One possible explanation for the failure to infect mice is a low infectious load in the inoculum, which might be lower than the minimum required to produce an in vivo infection [52].

The difficulty of transmitting reindeer CWD to sheep was suggested earlier by an in vitro experiment, which demonstrated late amplification of reindeer prions in sheep brain substrates [22]. Further adaptation of this prion disease in sheep through serial passages may lead to a more stable and aggressive prion disease, as seen in similar experiments with mule deer CWD [32], serial passages of Norwegian CWD prions in transgenic and gene-targeted mice [18], and serial passages of BSE in mice [53]. The concept of prion adaptation in different hosts and transgenic mice has also been demonstrated in studies with sheep-passaged elk CWD [54] and white-tailed deer-passaged scrapie prions [55]. Alternatively, this transmission of reindeer CWD to sheep might represent an example of non-adaptive prion amplification, and prions may fail to propagate upon further passages in sheep [56, 57].

We observed some discrepancies between the amplification techniques, with RT-QuIC being more sensitive than PMCA for detecting prions. Similar conditions are observed in the case of human prion diseases where the classical RT-QuIC is sensitive for detecting sporadic Creutzfeldt-Jakob disease (CJD) prions but not variant CJD, while the opposite is true for PMCA [58–60]. One important aspect to consider is that both techniques employed different reaction substrates (recombinant BvPrP_90-231_ protein for RT-QuIC and VRQ/VRQ sheep brain substrate for PMCA), which might explain this discrepancy. At this stage, we cannot rule out the possibility that in this experiment, sheep may propagate prions with unusual properties that are difficult to detect. These prions could be detected with varying degrees of efficiency by both techniques, depending on the reaction substrate used. However, correlation between these two techniques is not always achievable, especially under experimental settings such as ours, where for obvious reasons, reindeer CWD in sheep controls was lacking. Another challenge derived from the lack of validated methods is the examination of tissues with ELISA. The available commercial ELISA kits have been validated for scrapie, BSE and CWD but not for prions transmitted to a new species [17]. This calls for caution when interpreting these test results. In the RPLN of sheep 90542, the OD was above the cut-off for CWD and BSE but below that for scrapie. The OD values of two other lymph nodes were just below the cut-off (borderline negative) but were much higher than those of the remaining tissues and correlated with RT-QuIC seeding activity. It is not unlikely that these equivocal results represent very low PrP^Sc^ in the examined samples [52].

In conclusion, our results document primary transmission of reindeer CWD to sheep by intracerebral inoculation, albeit with unusual disease characteristics. Given the difficulty of transmitting this strain to sheep, as judged by the long incubation period and the apparent low level of detectable prions, the risk for spillover of reindeer CWD to sheep is most likely low. These findings should be considered for risk assessment and further management of reindeer CWD. Despite this low risk, one must consider the possibility of subclinical carriers following transmission to sheep, as described for other interspecies transmissions, both natural and experimental [61–63]. This scenario presents potential hazards due to the challenges in identifying subclinical carriers. However, these carriers could be less likely to shed and transmit disease. Further oral inoculation studies in sheep will be valuable to support the findings of the present study.

### Supplementary Information

Additional file 1.** Medication and dosage regime for the inoculation process and the recovery period**.

Additional file 2.** Analysis of rectoanal mucosa-associated lymphoid tissue (RAMALT) homogenates at different time points by protein misfolding cyclic amplification (PMCA)**. Western blot (WB) of the 5th PMCA round is shown. No PrP^Sc^ was detected in the RAMALT collected at different time points (12, 18, 24, 30, and 36 mpi) from inoculated sheep. RAMALT samples from healthy and classical scrapie (c. scrapie) sheep were added as negative and positive controls, respectively. The numbers to the right of each WB indicate the molecular weight marker in kilodaltons (kDa). *RH* ramalt homogenate, *PK* proteinase K, *mpi* months post-inoculation.

Additional file 3. **Lymphoid follicular (LF) count of recto-anal mucosa-associated lymphoid tissue (RAMALT) biopsies for all the acquired samples during the experiment.** The LF count for each individual sample is provided below the graph. The average LF count from all animals at each time point is displayed within the graph. Each animal is represented by a unique colour, while the average LF count is depicted in dark blue. LF counts did not decrease over time, and the number of LFs varied between animals and across different time points. Only one sample contained no LFs.

Additional file 4.** Brain vacuolation scores of inoculated sheep and non-inoculated sheep**. Heatmap representation of vacuolation scores in five grey matter regions (to the left of the bold line) and four white matter regions (to the right of the bold line). In the obex section, the dorsal motor nucleus of the vagus nerve, hypoglossal nucleus, accessory cuneate nucleus and olivary nucleus were included in the evaluation. Pons and cerebellar peduncles were evaluated in a transverse section cut through the cerebellum and the brainstem at the level of the caudal cerebellar peduncle. The areas included in the evaluation were the lateral vestibular nucleus, the trigeminal nucleus and the reticular formation. The mesencephalon was evaluated in a section through the superior colliculus and included the central grey matter, the red nucleus and the substantia nigra. Central white matter in the cerebellum was evaluated in a sagittal section taken at the midline of the cerebellum. The thalamus, corpus callosum and capsula interna were evaluated in a transverse section through the piriform lobe, which included the hippocampus. An area of the cerebral cortex was scored in a section taken at the ansate sulcus. *WM* white matter, *CNS* central nervous system.

Additional file 5.** Trace element analysis of liver and serum**.

Additional file 6.** Western blot of tissues following 6 rounds of PMCA. ** The figure shows 65 tissues analysed with PMCA in the study. Five tissue samples were positive after 6 rounds of amplification. Six spleen samples (one from each animal) were also included. *PK* proteinase K, *C. Scrapie* classical scrapie.

Additional file 7.** Summary of the results obtained by PMCA, RT-QuIC, western blot and ELISA**.

Additional file 8.** Conventional western blot of the CWD isolates used as inoculum and two lymph nodes of the inoculated sheep**. A. Western blots (TeSeE) from the brain of the two reindeer CWD donors (17-CD11089 and 18-CD2788) constituting the inoculum together with a control sheep with classical scrapie and a negative control. B. Western blots (TeSeE) from the DJLN of sheep 90507 and the PLN of sheep 90501, together with a control sheep with classical scrapie. The two panels were extracted from two different blots. *DJLN* distal jejunal lymph node, *PLN* parotid lymph node, *kDa* molecular mass in kilodaltons.

## Data Availability

Data supporting the conclusions are included within the paper and the additional files.
