# Supplementary material for: Transmission of Norwegian reindeer CWD to sheep by intracerebral inoculation results in an unusual phenotype and prion distribution
Source: Vet Res. 2024 Jul 29;55:94. doi: 10.1186/s13567-024-01350-6 (PMC11285437; doi:10.1186/s13567-024-01350-6)
Supplement: Supplementary file 1 — Additional file 1. Medication and dosage regime for the inoculation process and the recovery period. [file 13567_2024_1350_MOESM1_ESM.docx]

|  | **Drug** | **Dose** | **Administration** | **Treatment duration** |
| --- | --- | --- | --- | --- |
| **General anesthesia** | Tiletamine and zolazepam mixture | 7.5 mg/kg (initial) | Intramuscularly | Inoculation process |
|  |  | 1-2 mg/kg (supplemental anesthesia) |  |  |
| **Local anesthesia** | Lidocaine hydrochloride, adrenaline tartrat | 2 mL | Subcutaneously | Inoculation process |
| **Analgesia** | Flunixin meglumine | 2.2 mg/kg | Intravenously | 3 days |
| **Prophylactic antimicrobial treatment** | Crystalline penicillin | 100 000 IU/kg | Intravenously | During the procedure |
|  | Procaine benzylpenicillin | 60 mg/kg | Intramuscularly | 3 days |
|  | Oxytetracycline | 10 mg/kg | Intravenously | 5 days |
