# Supplementary figures and images for: Transmission of Norwegian reindeer CWD to sheep by intracerebral inoculation results in an unusual phenotype and prion distribution
Source: Vet Res. 2024 Jul 29;55:94. doi: 10.1186/s13567-024-01350-6 (PMC11285437; doi:10.1186/s13567-024-01350-6)

# RHs - 12 mpi

# RHs - 18 mpi

# RHs - 24 mpi

90501  
90506  
90507  
90525  
90542  
90530

+

90501  
90506  
90507  
90525  
90542

+

90501  
90506  
90507  
90525  
90542

+

PK  
50µg/mL  
28kDa

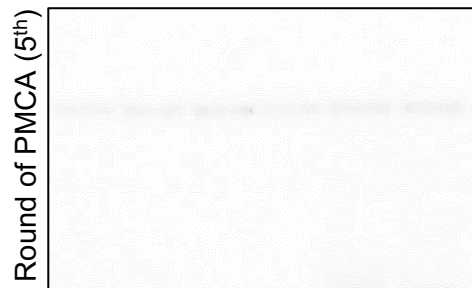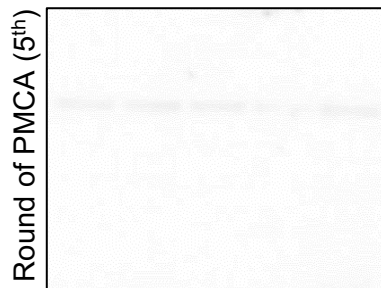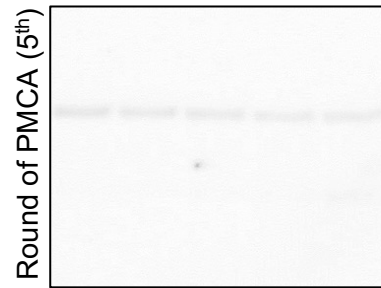

# RHs - 30 mpi

# RHs - 36 mpi

RH healthy sheep  
RH c. scrapie sheep

90501  
90506  
90507  
90525  
90542

+

90501  
90506  
90507  
90525

+

PK  
50µg/mL  
28kDa

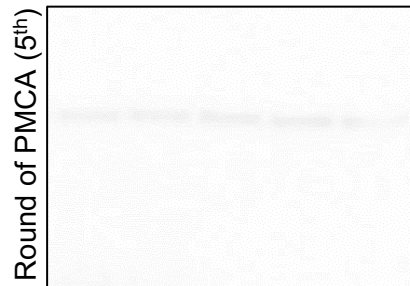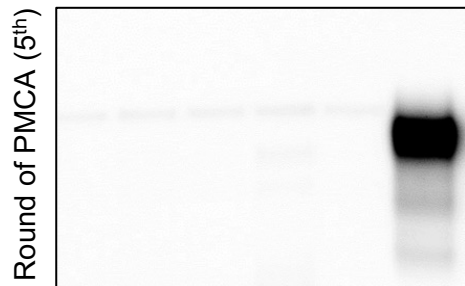

Supplement: Supplementary file 2 — Additional file 2. Analysis of rectoanal mucosa-associated lymphoid tissue (RAMALT) homogenates at different time points by protein misfolding cyclic amplification (PMCA). Western blot (WB) of the 5th PMCA round is shown. No PrPSc was detected in the RAMALT collected at different time points (12, 18, 24, 30, and 36 mpi) from inoculated sheep. RAMALT samples from healthy and classical scrapie (c. scrapie) sheep were added as negative and positive controls, respectively. The numbers to the right of each WB indicate the molecular weight marker in kilodaltons (kDa). RH ramalt homogenate, PK proteinase K, mpi months post-inoculation. [file 13567_2024_1350_MOESM2_ESM.pdf]

RAMALT LF count

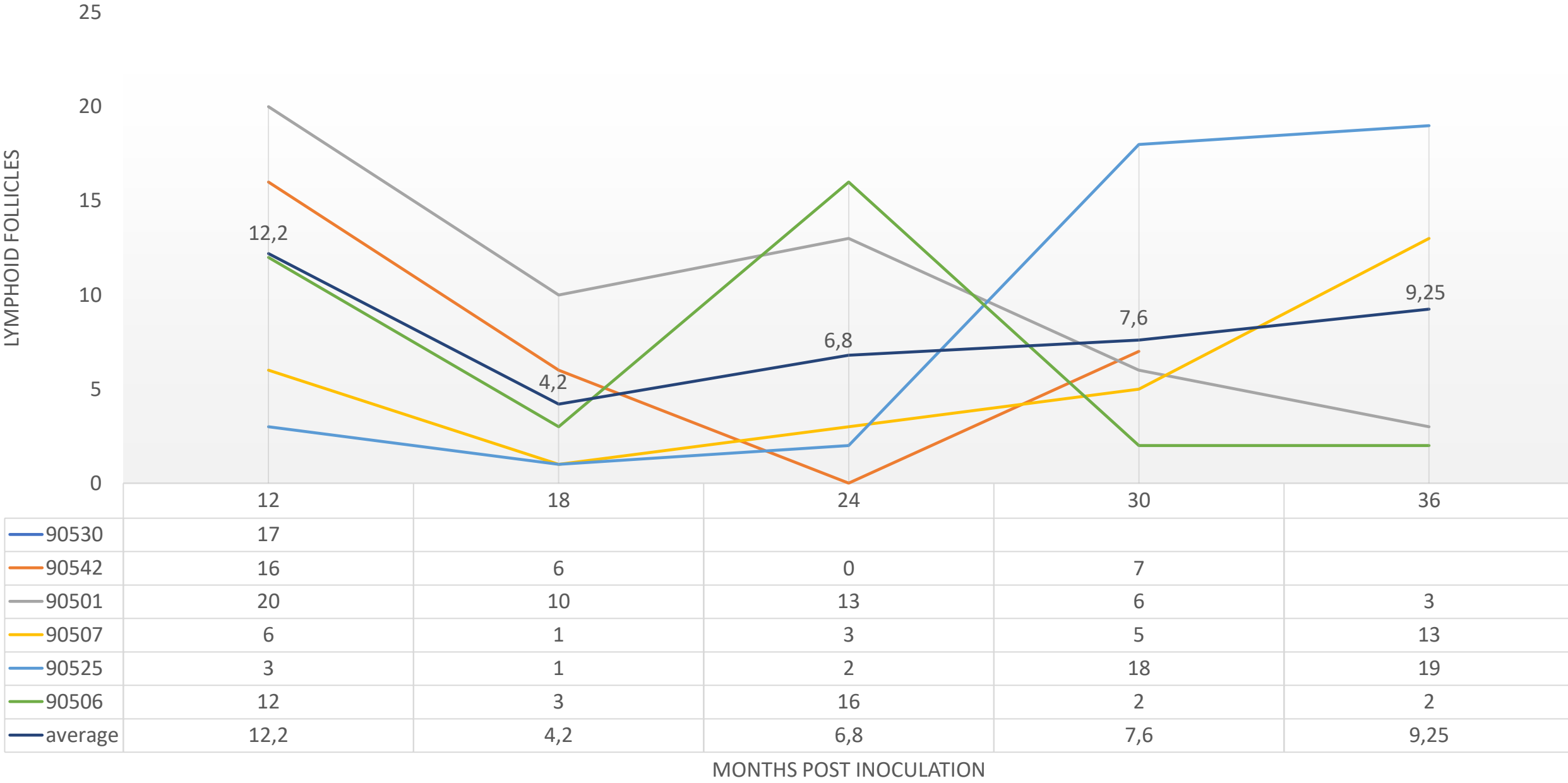

90530 90542 90501 90507 90525 90506 average

Supplement: Supplementary file 3 — Additional file 3. Lymphoid follicular (LF) count of recto-anal mucosa-associated lymphoid tissue (RAMALT) biopsies for all the acquired samples during the experiment. The LF count for each individual sample is provided below the graph. The average LF count from all animals at each time point is displayed within the graph. Each animal is represented by a unique colour, while the average LF count is depicted in dark blue. LF counts did not decrease over time, and the number of LFs varied between animals and across different time points. Only one sample contained no LFs. [file 13567_2024_1350_MOESM3_ESM.pdf]

A

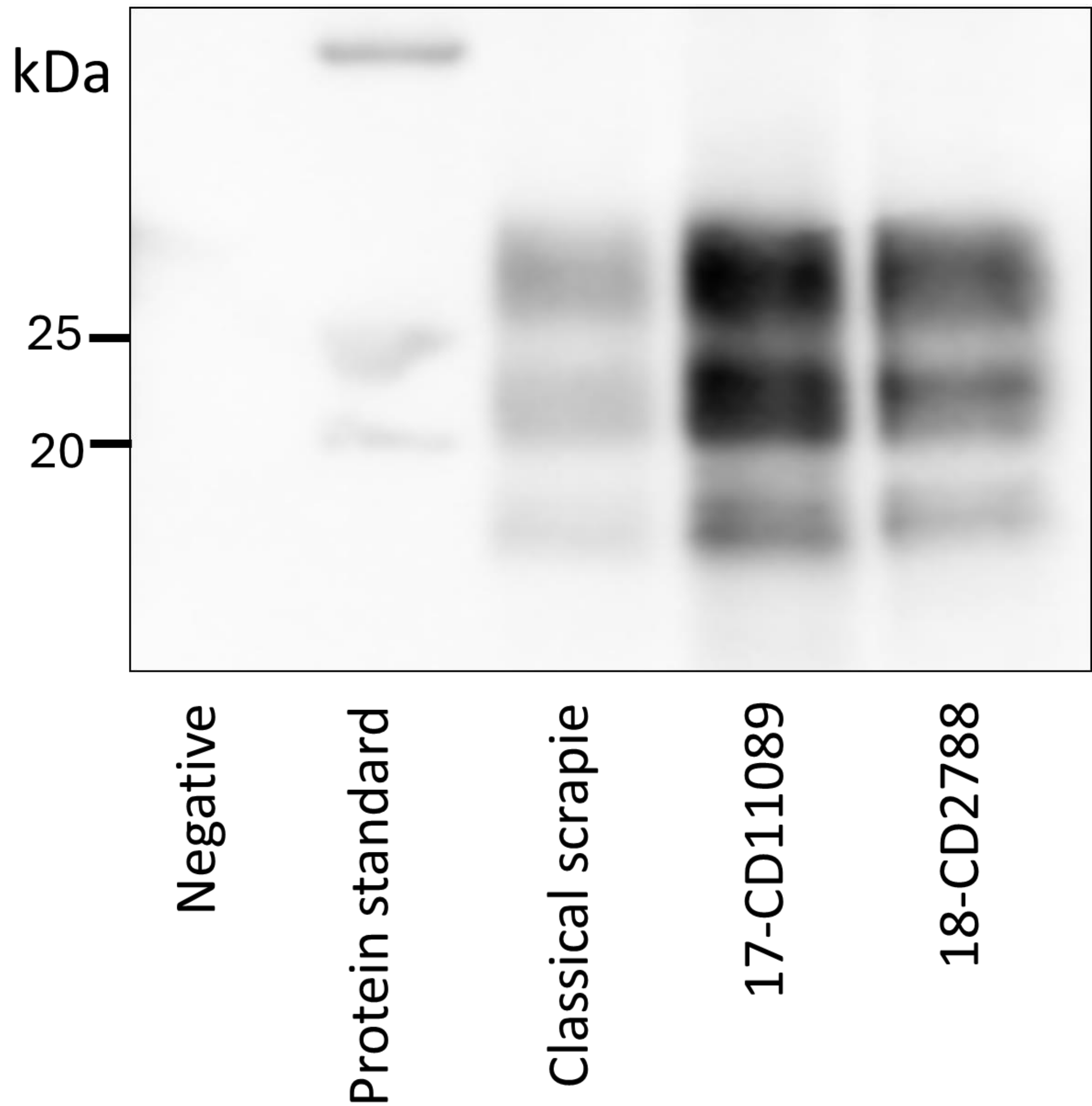

B

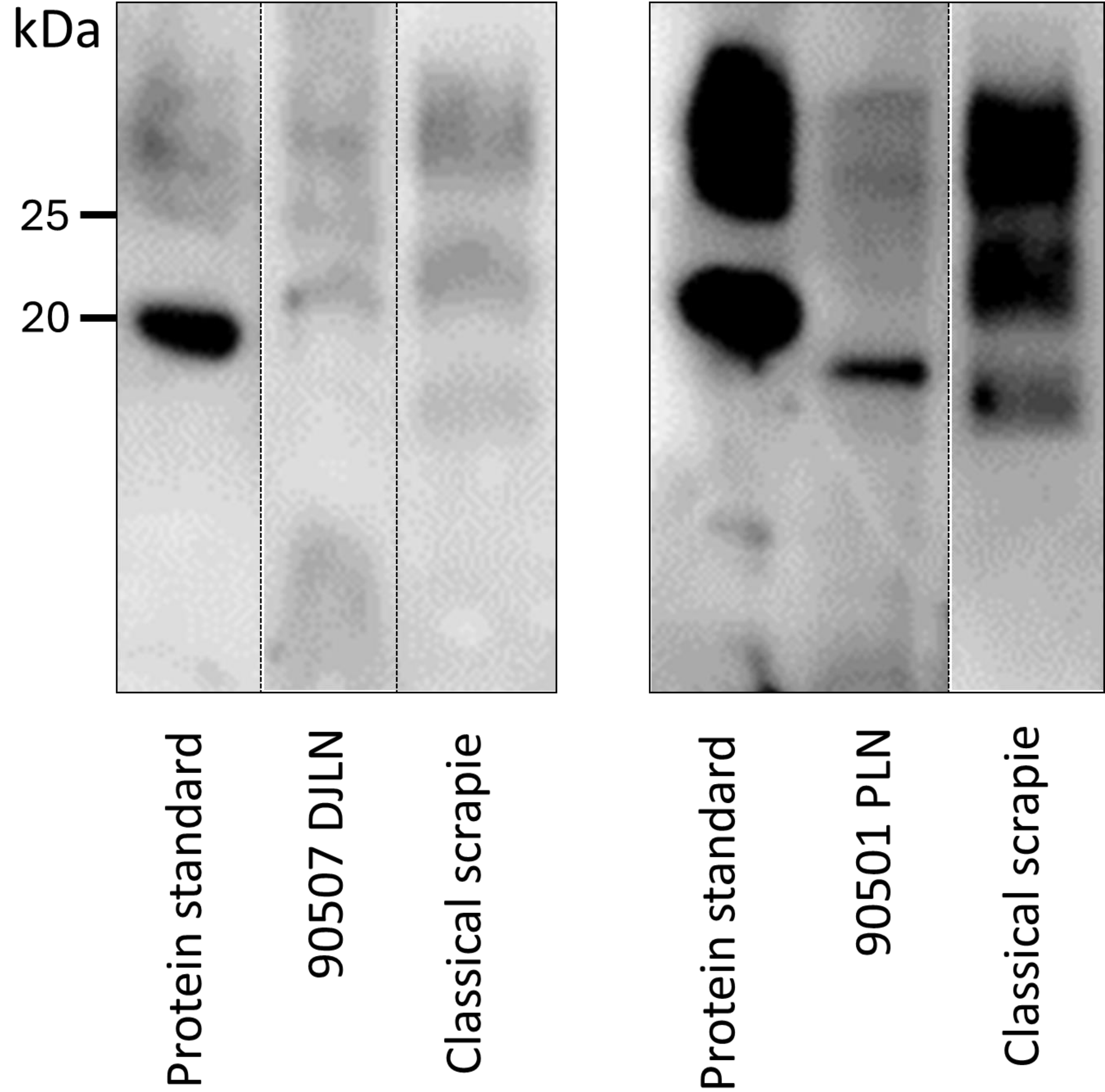

Supplement: Supplementary file 8 — Additional file 8. Conventional western blot of the CWD isolates used as inoculum and two lymph nodes of the inoculated sheep. A. Western blots (TeSeE) from the brain of the two reindeer CWD donors (17-CD11089 and 18-CD2788) constituting the inoculum together with a control sheep with classical scrapie and a negative control. B. Western blots (TeSeE) from the DJLN of sheep 90507 and the PLN of sheep 90501, together with a control sheep with classical scrapie. The two panels were extracted from two different blots. DJLN distal jejunal lymph node, PLN parotid lymph node, kDa molecular mass in kilodaltons. [file 13567_2024_1350_MOESM8_ESM.pdf]
