# Supplementary material for: Transmission of Norwegian reindeer CWD to sheep by intracerebral inoculation results in an unusual phenotype and prion distribution
Source: Vet Res. 2024 Jul 29;55:94. doi: 10.1186/s13567-024-01350-6 (PMC11285437; doi:10.1186/s13567-024-01350-6)
Supplement: Supplementary file 4 — Additional file 4. Brain vacuolation scores of inoculated sheep and non-inoculated sheep. Heatmap representation of vacuolation scores in five grey matter regions (to the left of the bold line) and four white matter regions (to the right of the bold line). In the obex section, the dorsal motor nucleus of the vagus nerve, hypoglossal nucleus, accessory cuneate nucleus and olivary nucleus were included in the evaluation. Pons and cerebellar peduncles were evaluated in a transverse section cut through the cerebellum and the brainstem at the level of the caudal cerebellar peduncle. The areas included in the evaluation were the lateral vestibular nucleus, the trigeminal nucleus and the reticular formation. The mesencephalon was evaluated in a section through the superior colliculus and included the central grey matter, the red nucleus and the substantia nigra. Central white matter in the cerebellum was evaluated in a sagittal section taken at the midline of the cerebellum. The thalamus, corpus callosum and capsula interna were evaluated in a transverse section through the piriform lobe, which included the hippocampus. An area of the cerebral cortex was scored in a section taken at the ansate sulcus. WM white matter, CNS central nervous system. [file 13567_2024_1350_MOESM4_ESM.pdf]

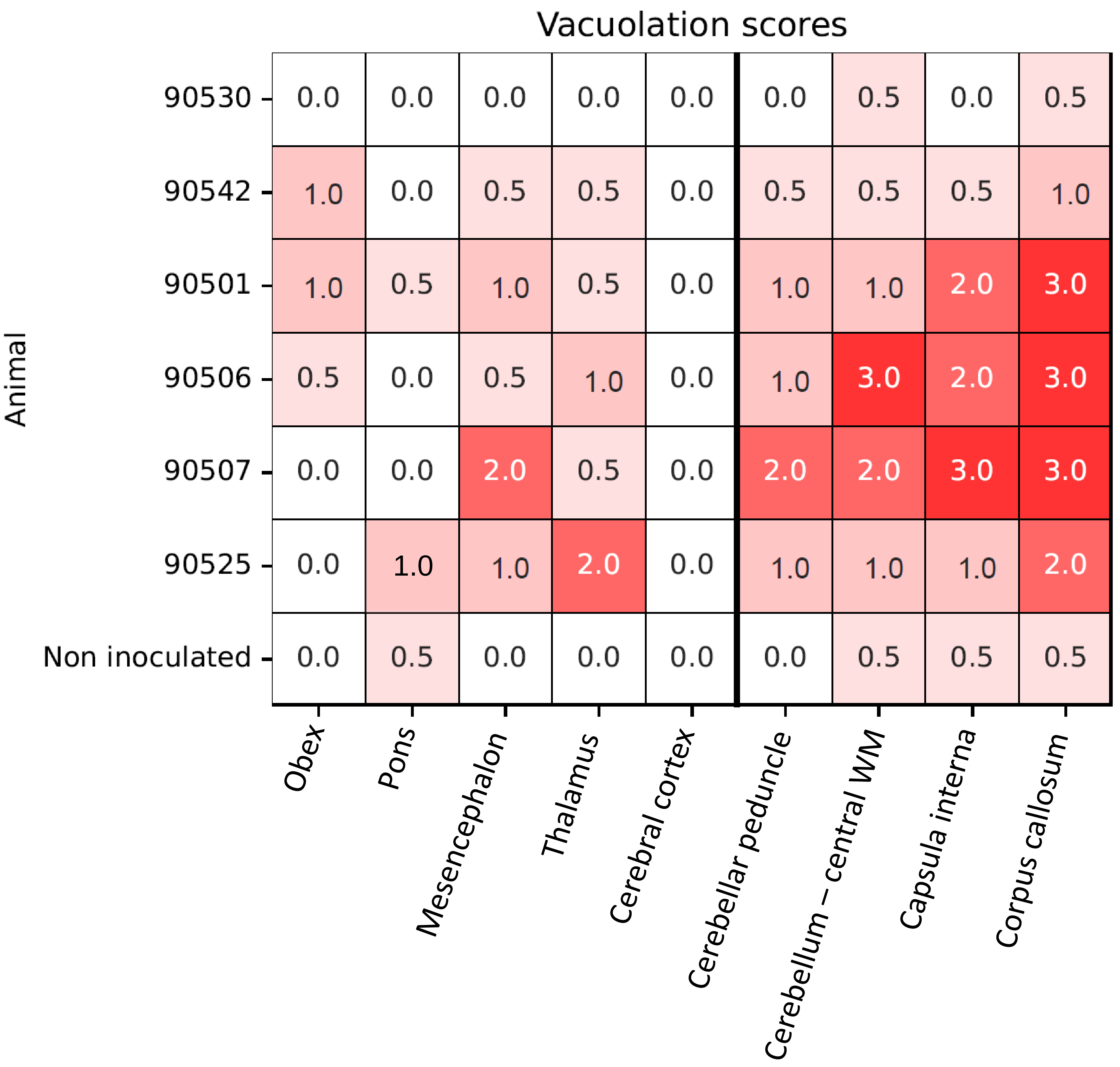
Vacuolation scores
Animal
90530
90542
90501
90506
90507
90525
Non inoculated
0.0 0.0 0.0 0.0 0.0 0.0 0.5 0.0 0.5
1.0 0.0 0.5 0.5 0.0 0.5 0.5 0.5 1.0
1.0 0.5 1.0 0.5 0.0 1.0 1.0 2.0 3.0
0.5 0.0 0.5 1.0 0.0 1.0 3.0 2.0 3.0
0.0 0.0 2.0 0.5 0.0 2.0 2.0 3.0 3.0
0.0 1.0 1.0 2.0 0.0 1.0 1.0 1.0 2.0
0.0 0.5 0.0 0.0 0.0 0.0 0.5 0.5 0.5
Obex
Pons
Mesencephalon
Thalamus
Cerebral cortex
Cerebellar peduncle
Cerebellum – central WM
Capsula interna
Corpus callosum
CNS region
