# Supplementary material for: Transmission of Norwegian reindeer CWD to sheep by intracerebral inoculation results in an unusual phenotype and prion distribution
Source: Vet Res. 2024 Jul 29;55:94. doi: 10.1186/s13567-024-01350-6 (PMC11285437; doi:10.1186/s13567-024-01350-6)
Supplement: Supplementary file 5 — Additional file 5. Trace element analysis of liver and serum. [file 13567_2024_1350_MOESM5_ESM.pdf]

Animal	Liver							Serum	
	Concentration in mg/kg (wet mass) (Reference range)						Age (months)	Concentration in umol/l (Reference range)	Age (months)
	Fe (35-350)	Co (0.025-0.1)	Cu (6-150)	Se (0.02-25)	Mo	Pb (0-4.1)		Cu (9-25)	
**90530**	140	0.059	24	0.39	0.85	<0.02	20	22.8	20
**90542**	130	0.051	58	0.41	1.4	0.034	34	17	32
**90525**	140	0.058	37	0.42	1.2	<0.02	45	13.7	39
**90506**	65	0.071	49	<0.009	1.2	<0.02	45	16.7	32
**90507**	120	0.053	120	0.48	1.1	<0.02	45	18.7	32
**90501**	180	<0.02	87	0.30	0.57	<0.02	45	15.5	32
