# Supplementary material for: Transmission of Norwegian reindeer CWD to sheep by intracerebral inoculation results in an unusual phenotype and prion distribution
Source: Vet Res. 2024 Jul 29;55:94. doi: 10.1186/s13567-024-01350-6 (PMC11285437; doi:10.1186/s13567-024-01350-6)
Supplement: Supplementary file 6 — Additional file 6. Western blot of tissues following 6 rounds of PMCA. The figure shows 65 tissues analysed with PMCA in the study. Five tissue samples were positive after 6 rounds of amplification. Six spleen samples (one from each animal) were also included. PK proteinase K, C. Scrapie classical scrapie. [file 13567_2024_1350_MOESM6_ESM.pdf]

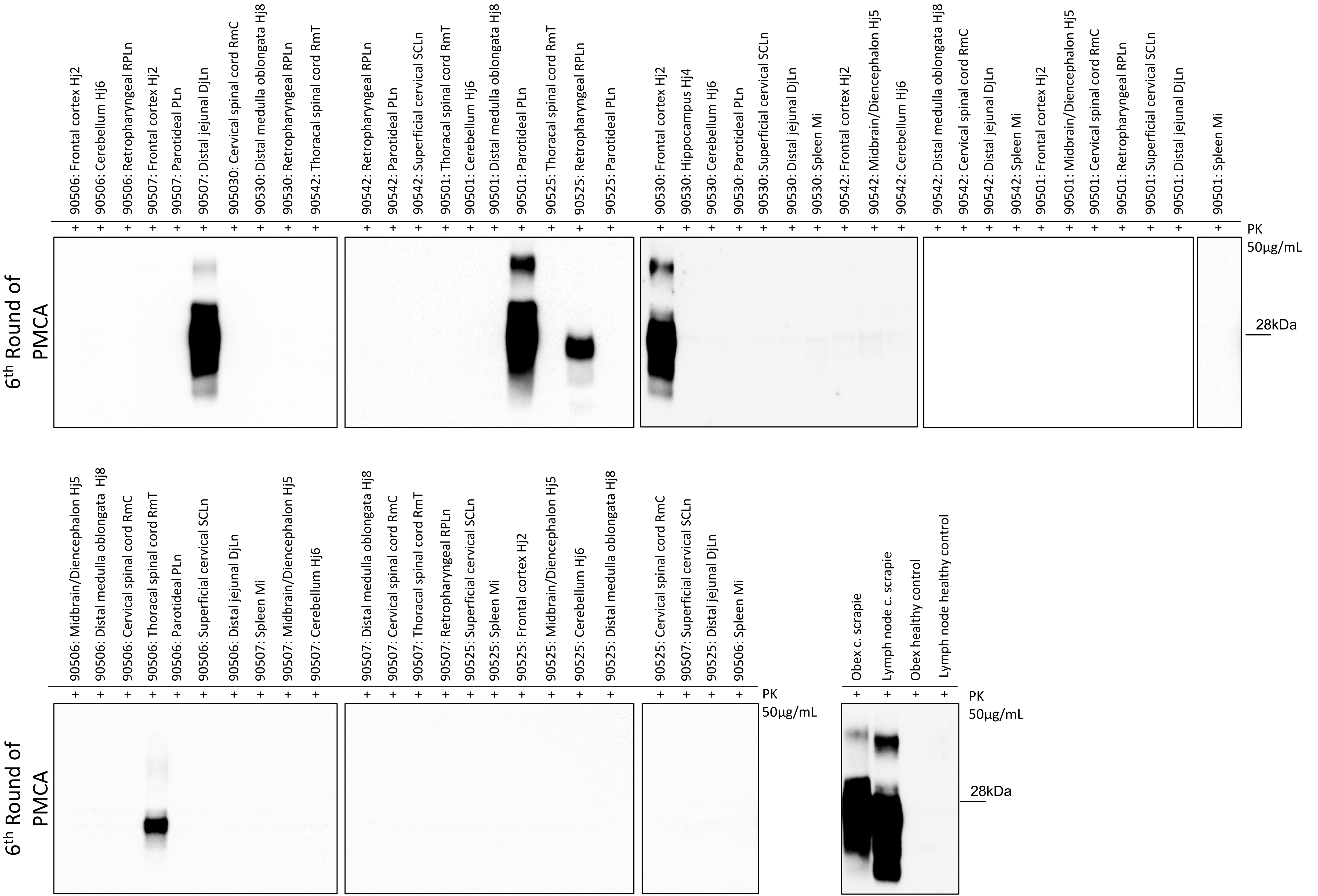
6th Round of PMCA
90506: Frontal cortex Hj2
90506: Cerebellum Hj6
90506: Retropharyngeal RPLn
90507: Frontal cortex Hj2
90507: Parotideal PLn
90507: Distal jejunal DjLn
90530: Cervical spinal cord RmC
90530: Distal medulla oblongata Hj8
90530: Retropharyngeal RPLn
90542: Thoracal spinal cord RmT
90542: Retropharyngeal RPLn
90542: Parotideal PLn
90542: Superficial cervical SCLn
90501: Thoracal spinal cord RmT
90501: Cerebellum Hj6
90501: Distal medulla oblongata Hj8
90501: Parotideal PLn
90525: Thoracal spinal cord RmT
90525: Retropharyngeal RPLn
90525: Parotideal PLn
90530: Frontal cortex Hj2
90530: Hippocampus Hj4
90530: Cerebellum Hj6
90530: Parotideal PLn
90530: Superficial cervical SCLn
90530: Distal jejunal DjLn
90530: Spleen Mi
90542: Frontal cortex Hj2
90542: Midbrain/Diencephalon Hj5
90542: Cerebellum Hj6
90542: Distal medulla oblongata Hj8
90542: Cervical spinal cord RmC
90542: Distal jejunal DjLn
90542: Spleen Mi
90501: Frontal cortex Hj2
90501: Midbrain/Diencephalon Hj5
90501: Cervical spinal cord RmC
90501: Retropharyngeal RPLn
90501: Superficial cervical SCLn
90501: Distal jejunal DjLn
90501: Spleen Mi
PK 50µg/mL
28kDa
6th Round of PMCA
90506: Midbrain/Diencephalon Hj5
90506: Distal medulla oblongata Hj8
90506: Cervical spinal cord RmC
90506: Thoracal spinal cord RmT
90506: Parotideal PLn
90506: Superficial cervical SCLn
90506: Distal jejunal DjLn
90507: Spleen Mi
90507: Midbrain/Diencephalon Hj5
90507: Cerebellum Hj6
90507: Distal medulla oblongata Hj8
90507: Cervical spinal cord RmC
90507: Thoracal spinal cord RmT
90507: Retropharyngeal RPLn
90525: Superficial cervical SCLn
90525: Spleen Mi
90525: Frontal cortex Hj2
90525: Midbrain/Diencephalon Hj5
90525: Cerebellum Hj6
90525: Distal medulla oblongata Hj8
90525: Cervical spinal cord RmC
90507: Superficial cervical SCLn
90525: Distal jejunal DjLn
90506: Spleen Mi
PK 50µg/mL
Obex c. scrapie
Lymph node c. scrapie
Obex healthy control
Lymph node healthy control
PK 50µg/mL
28kDa
