# Supplementary material for: Transmission of Norwegian reindeer CWD to sheep by intracerebral inoculation results in an unusual phenotype and prion distribution
Source: Vet Res. 2024 Jul 29;55:94. doi: 10.1186/s13567-024-01350-6 (PMC11285437; doi:10.1186/s13567-024-01350-6)
Supplement: Supplementary file 7 — Additional file 7. Summary of the results obtained by PMCA, RT-QuIC, western blot and ELISA. [file 13567_2024_1350_MOESM7_ESM.docx]

| Animal | Technique | Brain | | | | | | Spinal cord | | | Peripheral | | | | |
| --- | --- | --- | --- | --- | --- | --- | --- | --- | --- | --- | --- | --- | --- | --- | --- |
|  |  | Olfactory bulb | Frontal cortex | Diencephelon | Hippocampus | Cerebellum | Obex | Cervical | Thoracal | Lumbar | PLN | SCLN | RPLN | DJLN | Spleen |
| 90530 | ELISA | NA | NA | NA | NA | NA | - | - | - | NA | - | NA | - | - | - |
|  | RT-QuIC | - | - | NA | - | + | + | + | NA | NA | - | + | + | + | NA |
|  | PMCA | NA | + | - | - | - | - | - | NA | NA | - | - | - | - | - |
|  | WB | NA | NA | NA | NA | NA | NA | - | NA | NA | - | NA | - | NA | - |
| 90542 | ELISA | NA | NA | NA | NA | NA | - | NA | NA | NA | - | NA | *0.192* | - | - |
|  | RT-QuIC | NA | - | - | NA | - | - | - | + | - | + | + | + | + | NA |
|  | PMCA | NA | - | - | NA | - | - | - | - | NA | - | - | - | - | - |
|  | WB | NA | NA | NA | NA | NA | NA | NA | - | NA | - | NA | - | - | NA |
| 90501 | ELISA | NA | - | NA | NA | NA | - | NA | - | - | - | - | - | - | - |
|  | RT-QuIC | + | - | - | - | + | + | - | + | NA | + | + | + | + | NA |
|  | PMCA | NA | - | - | NA | - | - | - | - | NA | + | - | - | - | - |
|  | WB | NA | NA | NA | NA | NA | NA | NA | - | NA | + | - | NA | NA | - |
| 90506 | ELISA | NA | - | NA | NA | NA | - | NA | - | - | - | - | - | - | - |
|  | RT-QuIC | - | + | - | - | + | - | - | - | NA | - | - | + | - | NA |
|  | PMCA | NA | - | - | NA | - | - | - | - | NA | - | - | - | - | - |
|  | WB | NA | NA | NA | NA | NA | NA | NA | NA | NA | - | NA | NA | NA | - |
| 90507 | ELISA | NA | - | NA | NA | NA | - | NA | - | - | - | - | - | - | - |
|  | RT-QuIC | - | - | - | - | - | - | - | - | NA | + | + | - | + | NA |
|  | PMCA | NA | - | - | NA | - | - | - | - | NA | - | - | - | + | - |
|  | WB | NA | NA | - | NA | NA | NA | NA | - | NA | - | NA | - | + | - |
| 90525 | ELISA | NA | - | NA | NA | NA | - | NA | - | - | *0.141* | *0.133* | - | - | - |
|  | RT-QuIC | - | - | - | - | - | - | - | + | NA | + | + | + | + | NA |
|  | PMCA | NA | - | - | NA | - | - | - | + | NA | - | - | + | - | - |
|  | WB | NA | NA | NA | NA | NA | NA | NA | - | NA | - | - | - | NA | - |

+ – positive - – negative, NA – not performed. Number in italic indicates the optical density (OD) value of the respective tissue. Value is only provided for tissues with OD above 0.1. DJLN – distal jejunal lymph node; RPLN – medial retropharyngeal lymph node; SCLN – superficial cervical lymph node; PLN – parotid lymph node.
